# Clinical Factors Associated with Asymptomatic Women Having Inconclusive Screening Mammography Results: Experiences from a Single Medical Center in Taiwan

**DOI:** 10.3390/ijerph18105410

**Published:** 2021-05-19

**Authors:** Chun-Li Wang, Pi-Shan Hsu, Chia-Yen Lin, Shun-Fa Yang

**Affiliations:** 1 Institute of Medicine, Chung Shan Medical University, Taichung 40201, Taiwan; chun_li@vghtc.gov.tw; 2 Department of Family Medicine, Taichung Veterans General Hospital, Taichung 40705, Taiwan; pshsu3@gmail.com; 3 Graduate Institute of Microbiology and Public Health, College of Veterinary Medicine, National Chung-Hsing University, Taichung 40227, Taiwan; 4 Division of Urology, Department of Surgery, Taichung Veterans General Hospital, Taichung 40705, Taiwan; 5 Department of Medical Research, Chung Shan Medical University Hospital, Taichung 40705, Taiwan

**Keywords:** mammography, BI-RADS 0, breast cancer screening, preventive medicine

## Abstract

Screening mammography is used worldwide for the early detection of breast cancer in women experiencing no symptoms. The Breast Imaging Reporting and Database System (BI-RADS) is used to report mammographic findings. However, little is known about the clinical characteristics of Asian women with BI-RADS category 0, and we aimed to explore such characteristics in the context of Taiwan. This retrospective cross-sectional study was conducted using data from a single tertiary medical center. We examined the association of blood test data and estrogen exposure–related medical histories with BI-RADS reports from screening mammography of 4280 women between 1 January 2010 and 31 July 2019. The data of 4280 participants were evaluated, and they were categorized into BI-RADS category 0 (*n* = 413; 9.6%) and 1–5 (*n* = 3867; 90.4%) subgroups. In a multivariate analysis, breast surgery history and premenopausal status had a positive relationship with a category 0 status, with respective risk increases of 64% and 34% (*p* = 0.010 and 0.013). Hormone contraceptive use for ≥5 years was a negative independent predictor of having a category 0 status. In conclusion, breast surgery history and premenopausal status significantly increased the likelihood of individuals having incomplete mammographic findings, even when they were older than 45 years. Identifying related factors before screening mammography is helpful for clinical physicians to arrange more proper and alternative examination and obtain a definite diagnosis.

## 1. Introduction

Breast cancer is the third leading cause of cancer death among women in Taiwan. In 2017, approximately 13,965 women were diagnosed as having the disease, and more than 2300 women died of it. The rate of new cases of female breast cancer was 117.8 per 100,000 women per year and the mortality rate was 20.1 per 100,000 women per year. [1]. The updated recommendations of the US Preventive Services Task Force in 2016 suggest biennial screening mammography for women aged 50 to 74 years (B Recommendation) [2].

In Taiwan, the Health Promotion Administration policy on cancer screening stipulates free mammography for breast cancer screening once every two years for women aged 45 to 69 years or those aged 40 to 44 years whose second-degree relatives have had breast cancer. Radiologists then assess the mammography findings according to the American College of Radiology’s Breast Imaging Reporting and Data System (BI-RADS) [3,4]. This allows them to clearly and consistently communicate results to the referring physician. However, mammography results are sometimes incomplete, meaning that additional imaging evaluation (additional mammographic views or ultrasound) may be required; women with such incomplete results are considered to have a BI-RADS category of 0.

Several factors affect mammographic image quality. Technical factors include breast positioning, compression, optimum, sharpness, exposure, and contrast [5]. Clinical factors include a high mammographic breast density, menopausal status, and age [6]. Among such factors, high breast density, which reflects the extent of estrogen exposure, has the strongest association with several clinical characteristics and metabolic abnormalities, such as younger age, lower body mass index (BMI), premenopausal status, nulliparity, older age at first birth, family history of breast cancer [7], and high-density lipoprotein cholesterol (HDL-C), as a feature of metabolic syndrome [8].

Metabolic syndrome, a cluster of metabolic abnormalities including central obesity, dyslipidemia, insulin resistance, and hypertension, had been studied as a risk factor for several types of cancer [9,10,11,12,13]. Some prior studies indicated that women with metabolic syndrome were at elevated risk of developing breast cancer with worse prognosis, while others suggested the increased association was observed only in postmenopausal women, but not in premenopausal women [14]. Insulin resistance and chronic inflammation may be the underlying pathophysiology between metabolic syndrome and increased risk of breast cancer. However, the association between metabolic syndrome and breast density, as one of the major risk factors of breast cancer has been under-investigated. Whether a correlation between breast density and these other factors influences the interpretation of mammographic findings remains unclear.

This study investigated the association between factors, including patients’ clinical characteristics and metabolic abnormalities, especially metabolic syndrome components, and women having incomplete screening mammography examinations in Taiwan.

## 2. Materials and Methods

### 2.1. Study Population and Design

**Figure 1:**
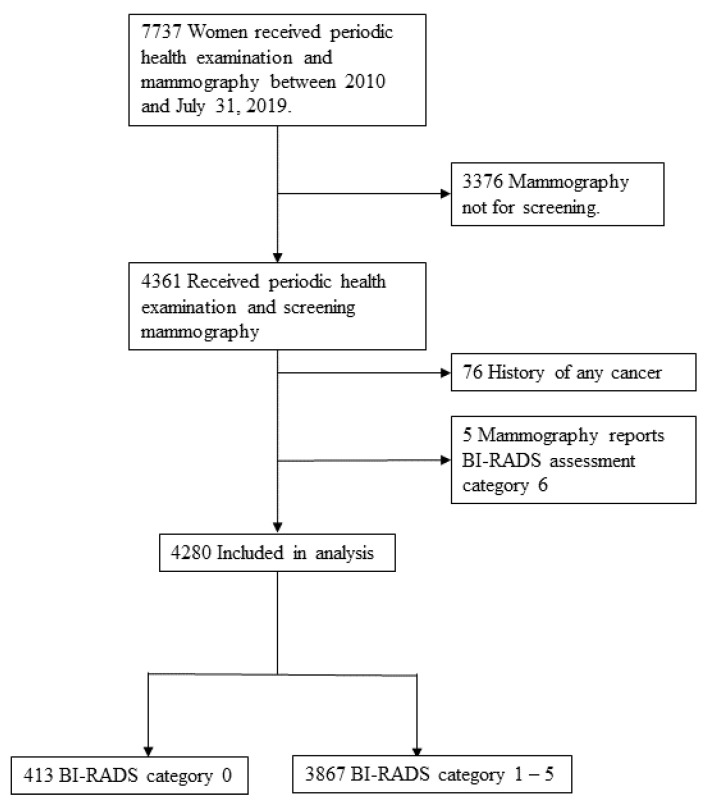
The flowchart of the study selection process.

The present study was retrospective and cross-sectional in design. The data of women who received both periodic health examinations and routine screening mammography under the National Health Insurance program at Taichung Veterans General Hospital from 1 January 2010 to 31 July 2019 were analyzed. Patients with a history of any cancer and those whose mammography reports revealed BI-RADS category 6 were excluded. To investigate the correlation between patient characteristics and BI-RADS categories, the patients were further divided into two subgroups (BI-RADS category 0 and BI-RADS category 1–5). The study selection process is presented in Figure 1. The study protocol was approved by the Institutional Review Board (IRB) of Taichung Veterans General Hospital (IRB number CE19332B).

### 2.2. Data Collection

The Health Promotion Administration of Taiwan provides a preventive care service package for adults aged 40 years and older. The package includes medical history taking, recording of health behaviors (smoking status, alcohol consumption, and exercise habit), physical examinations, and laboratory examinations of blood and urine samples. BMI was calculated as body weight (kg)/height^2^ (m^2^). The laboratory examinations of blood samples consisted of measurements of fasting glucose, triglycerides (TGs), total cholesterol, HDL-C, alanine and aspartate aminotransferases, creatinine, and estimated glomerular filtration rate. A person was determined to have metabolic syndrome if they satisfied three of the following criteria [15]: measured systolic blood pressure of ≥130 mmHg, diastolic blood pressure of ≥85 mmHg, or antihypertensive medication use; waist circumference of ≥80 cm; a TG level of ≥150 mg/dL or antihyperlipidemic medication use; fasting glucose of ≥100 mg/dL, oral antihyperglycemic drug use, or insulin injection administration; or HDL-C of <50 mg/dL.

We also compiled information on breast cancer risk factors, including history of a benign breast disease, menopausal status, history of breast surgery, history of breast cancer in second-degree relatives, age at menarche (<12, 12–13, or ≥14 years), age at first live birth (≤24, 25–29, or ≥30 years), number of abortions (0–1, ≥2), parity (nulliparous, one birth, or two or more births), breastfeeding history, and use of hormonal contraceptives (never, <5 years, or ≥5 years). Women who met one or more of the following conditions were considered in postmenopausal status: (1) self-report of natural menopause; (2) self-report of surgical menopause related to hysterectomy; or (3) self-report of surgical menopause related to bilateral oophorectomy. Those not meeting these conditions were considered in premenopausal status. Women who had lactated for at least 1 month were defined as having breastfed. The data were collected during interviews by trained staff before mammography.

### 2.3. Mammographic Assessment and Management Recommendations

As per the Health Promotion Administration policy on cancer screening, screening mammography is provided every 2 years for women aged 45 to 69 years and those aged 40 to 44 years whose second-degree relatives have had breast cancer. Mammography results were assessed with a single reading, and findings were recorded in a standardized lexicon by radiologists specializing in breast imaging by using the fourth or fifth edition of BI-RADS [3,4]. Individuals with normal, benign, or probably benign mammographic findings are interpreted as having BI-RADS category 1, 2 or 3. A BI-RADS category of 4 or 5 indicates a suspicion of malignancy, and category 6 indicates a biopsy-proven malignancy. Individuals with an incomplete assessment and for whom a practitioner has requested an additional diagnostic imaging survey are interpreted as having a BI-RADS category of 0.

On the basis of BI-RADS, the recommended management of positive mammography results in Taichung Veterans General Hospital is as follows [3,4]: Patients with a BI-RADS category of 2 have a 0% likelihood of malignancy and receive regular mammography screening every 2 years. Individuals with a BI-RADS category of 3 are associated with a low risk of breast cancer (>0% and ≤2% likelihood of malignancy), and repeated imaging in 6 months is recommended. A BI-RADS category of 4 or 5 necessitates an urgent pathologic evaluation with biopsy. A BI-RADS category of 0 indicates that additional diagnostic mammography or ultrasonography is required as soon as possible to provide a final assessment.

Breast composition, as known as breast density, is also categorized to four groups according to ACR-BIRADS classification. The new breast composition categories are as follows [4]: (A) the breasts are almost entirely fatty, (B) there are scattered areas of fibroglandular density, (C) the breasts are heterogeneously dense, which may obscure small masses, (D) the breasts are extremely dense, which lowers the sensitivity of mammography.

### 2.4. Statistical Analysis

Categorical data were expressed as numbers and percentages, whereas continuous variables were expressed as means ± standard deviations. Associations between all categorical variables and BI-RADS categories were determined through the chi-squared, Fisher’s exact, and the Mann–Whitney U test. We built our analysis model with a backward logistic regression model. Potential risk factors in the univariate logistic regression with *p* values less than 0.100 were entered into the multivariate model. A two-sided *p* value of <0.05 was considered statistically significant. All analyses were performed using SPSS version 22.0 (International Business Machines Corp., New York, NY, USA).

## 3. Results

In total, 4280 women received both periodic health examinations and routine screening mammography at our hospital from 1 January 2010 to 31 July 2019. The average age of the participants was 56 years (range, 40–69) and 413 (9.6%) and 3867 (90.4%) patients were in the BI-RADS category 0 and 1–5 subgroups, respectively (Figure 1). Table 1 presents the clinical, reproductive, and laboratory parameters of those in the BI-RADS category subgroups.

The proportion of patients with premenopausal status was higher in the BI-RADS category 0 subgroup than in the category 1–5 subgroup (26.8% vs. 21.26%, respectively; *p* = 0.010). The incidence of breast surgery history in the two subgroups was 8.47% and 5.43%, respectively (*p* = 0.016). The proportion of individuals who underwent <2 abortions was higher in the BI-RADS category 0 subgroup than in the category 1–5 subgroup (82.32% vs. 77.79%; *p* = 0.039). A significantly smaller proportion of individuals in the BI-RADS category 0 group had used hormonal contraceptives for more than five years (1.94% vs. 4.45%; *p* = 0.048). Overall, most patients had high breast density category (BIRADS C-D) in both subgroups, but no significant difference between the two groups was noted.

Adjustment for baseline covariates (Table 2) revealed that the BI-RADS category 0 subgroup had a significantly lower association with hormonal contraceptive usage (OR = 0.91, 95% CI = 0.83–0.99, *p* = 0.034) and use of hormonal contraceptives for ≥5 years (OR = 0.42, 95% CI = 0.20–0.86, *p* = 0.018). Compared with the other subgroup, those in BI-RADS category 0 subgroup had a 33% (*p* = 0.034), 61% (*p* = 0.012), and 36% (*p* = 0.009) higher association with undergoing <2 abortions, having a history of breast surgery, and having premenopausal status, respectively. Based on multivariate analysis (Table 2), hormonal contraceptive use for ≥5 years (OR = 0.42; 95% CI, 0.21–0.87, *p* = 0.019) was a negative independent predictor of having an incomplete mammographic assessment. By contrast, a positive relationship was observed between BI-RADS category 0 and individuals having a history of breast surgery or premenopausal status.

## 4. Discussion

Despite the developing techniques of medical treatment of breast cancer, health examinations are a very important part of preventive medical care. Mammography is an established screening test for breast cancer, but the problem of incomplete assessment needs to be solved. The present study identified several factors leading to incomplete mammography findings. A breast surgery history and premenopausal status significantly increased the risk of individuals having incomplete results. Women with these features undergoing breast cancer screening may consider receiving initial ultrasound assessment instead.

Surgical interventions for the breast may be for cosmetic or health reasons, but most lead to breast tissue reposition and scar formation [16]. On mammography, scar tissue appears as an architectural distortion, tissue asymmetry, or spiculated opacity, affecting the interpretation of radiological findings. Mammography is inferior to ultrasonography in terms of distinguishing such postsurgical breast changes [17]; uncertain or false image interpretations are common. Therefore, women with a history of breast surgery are not suitable candidate to receive mammography as a breast cancer screening tool.

The association between mammographic sensitivity related to noncalcified lesions and BI-RADS breast density category is well known [3,4]. Premenopausal women, owing to their greater estrogen exposure, tend to have more fibroglandular breast tissue components and denser breasts; these factors reduce the accuracy and sensitivity of mammography [18,19,20,21,22]. The correlation between premenopausal status and having a BI-RADS category of 0 is in the same direction as the association with this category and breast density. High breast density, incomplete assessment, and the elevated breast cancer risk are interrelated. Beside premenopausal status, clinical factors such as increasing height, higher levels of education, and later first birth were associated with higher mammography density [23], and the change of mammography density in follow-up screening study was associated with breast cancer risk, reported recently in East Asian women [24]. Therefore, the national policy of only taking age and familial breast cancer history into consideration as criteria for women to undergo breast cancer screening in Taiwan may need to put more emphasis on the other clinical factors and arrange alternative and effective examination.

Exogenous hormone exposure is related to changes in breast density [25,26,27,28,29]. In most related studies, hormonal contraceptives, including those containing estrogen and progestin, increased individuals’ breast density on mammographic imaging. Our findings led to a different conclusion on the influence of hormonal contraceptive exposure. One previous study revealed that the use of estrogen alone for one year did not affect the mean mammographic breast density of women, but greater density was associated with the use of estrogen–progestin combination therapy [30]. Another study revealed an association of hormone exposure with mammographic density; among breast cancer–free individuals, oral contraceptive users had had a lower breast density than nonusers [29]. Other clinical factors are associated with the effects of exogenous hormone supplements on breast density changes, but a consensus has not been reached. Further details on oral contraceptive use (e.g., type) were not obtained in our study; further investigation of these factors may be warranted.

Regular health examinations slow down the transformation from preclinical phase to clinical phase, with a consequent reduction in mortality. A proper screening test must be acceptable to most of the population, simple, conveniently operatable, non-invasive, low danger, relatively low cost, and most important, must have a high sensitivity and specificity [31,32]. Mammography for breast cancer screening meets the criteria for a screening tool, and the reported sensitivity and specificity in Asian population were 77.0% and 91.4% [33]. As long as screening combined an adjunctive ultrasonography in specific women, the sensitivity and detection rate of early cancers increased [33,34,35,36]. Using an accurate screening tool for an appropriate population provides precise healthcare.

Though many studies have concluded that high breast density disturbs mammography assessment, more than 90% of the patients who underwent screening mammography in this study had complete finding, categorized as BI-RADS 1–5. This showed that the screening mammography examination in Taichung Veteran General Hospital could give enough assurance and confidence to the healthy women, and the results achieved superior performance compared to other developed countries [37]. This may result from the fact that Taichung Veteran General Hospital, as a medical center, has high resolution of image examination and high quality of reporting system by breast image radiologists. Breast density did not become a distracting factor.

The strengths of this study are as follows: first, the study contributes to the literature by examining the factors related to incomplete mammography screening, serving to remind clinicians to use the most suitable and sensitive tools for breast cancer screening. Second, our study had a large sample size and a long recruitment period, and all patients had comprehensive blood and reproductive data. Nevertheless, this study has limitations. This was a retrospective single-center study, limiting the generalizability of our results. Furthermore, subsequent definite diagnosis and prognosis after incomplete mammography, which are among the most vital concerns for clinicians, were not traced in our study. Finally, as mentioned, owing to different breast density changes that can result from different hormone supplement regimens and frequencies, more details on oral contraceptive treatments should be obtained.

## 5. Conclusions

The completion of screening mammography was determined mostly by patients’ medical history and estrogen exposure background rather than by metabolic syndrome–related factors. Our results indicate that breast surgery history and premenopausal status are independently associated with individuals having a BI-RADS category of 0. Identifying related factors and providing timely health education are crucial so that additional examinations can be arranged to obtain a definite diagnosis as early as possible.

## Figures and Tables

**Table 1:** Clinical characteristics and component of metabolic syndrome of participants by BI-RADS category.

	BI-RADS Category 1–5 (*n* = 3867)		BI-RADS Category 0 (*n* = 413)		*p* Value
Age	56.92	±7.20	56.30	±7.51	0.124
Metabolic syndrome					0.978
No	2985	(77.19%)	318	(77.00%)	
Yes	882	(22.81%)	95	(23.00%)	
WC ≥ 80 cm (*n* = 4270)	1571	(40.72%)	168	(40.78%)	1.000
TG ≥ 150 mg/dL (*n* = 4278)	3745	(96.90%)	401	(97.09%)	0.942
HDL < 50 mg/dL (*n* = 4270)	736	(19.08%)	79	(19.17%)	1.000
fasting glucose ≥ 100 mg/dL/diabetes (*n* = 4278) ^a^	3836	(99.25%)	411	(99.52%)	0.763
SBP ≥ 130 mmHg/DBP ≥ 85 mmHg/hypertension (*n* = 4278) ^a^	3860	(99.87%)	413	(100%)	1.000
BMI (*n* = 4276)					0.669
<18.5	110	(2.85%)	12	(2.91%)	
18.5–23.9	2040	(52.80%)	208	(50.49%)	
≥24	1714	(44.36%)	192	(46.60%)	
Current smoker	77	(1.99%)	10	(2.42%)	0.685
Alcohol drinking	379	(9.80%)	45	(10.90%)	0.534
Habit of exercise					0.368
No	1475	(38.14%)	147	(35.59%)	
<2.5 hr/week	1383	(35.76%)	162	(39.23%)	
≥2.5 hr/week	1009	(26.09%)	104	(25.18%)	
History of benign breast disease	714	(18.46%)	82	(19.85%)	0.533
Menopause status					0.010 *
Premenopausal	822	(21.26%)	111	(26.88%)	
Postmenopausal	3045	(78.74%)	302	(73.12%)	
History of breast surgery	210	(5.43%)	35	(8.47%)	0.016 *
Family history of breast cancer	278	(7.19%)	27	(6.54%)	0.698
Age at menarche, years					0.500
<12	104	(2.69%)	15	(3.63%)	
12–13	1342	(34.70%)	146	(35.35%)	
≥14	2421	(62.61%)	252	(61.02%)	
Age at first live birth, years (*n* = 3896)					0.428
≤24	1168	(33.20%)	125	(33.07%)	
25–29	1680	(47.75%)	171	(45.24%)	
≥30	670	(19.04%)	82	(21.69%)	
Abortion					0.039 *
0–1	3008	(77.79%)	340	(82.32%)	
≥2	859	(22.21%)	73	(17.68%)	
Parity					0.708
nulliparous	347	(8.97%)	35	(8.47%)	
1	419	(10.84%)	40	(9.69%)	
≥2	3101	(80.19%)	338	(81.84%)	
Breast feeding	2011	(52.00%)	233	(56.42%)	0.098
Use of hormonal contraceptives					0.048 *
No	3095	(80.04%)	343	(83.05%)	
<5 years	600	(15.52%)	62	(15.01%)	
≥5 years	172	(4.45%)	8	(1.94%)	
Breast composition BI-RADS					
A	23	(0.60%)	0	(0.00%)	0.182
B	138	(3.57%)	9	(2.18%)	
C	3324	(86.00%)	365	(88.38%)	
D	380	(9.83%)	39	(9.44%)	

Chi-Square test. ^a^ Fisher’s Exact test. Mann–Whitney U test. * *p* < 0.05. Values were expressed as numbers and percentages (or mean and SD).

**Table 2:** Univariate and multivariate logistic regression analysis: independent variables assessed incomplete mammographic evaluation (BI-RADS category 0).

		Univariate			Multivariate		
	Num	OR	95%CI	*p* Value	OR	95%CI	*p* Value
Metabolic syndrome							
No	318	ref.					
Yes	95	1.01	(0.79–1.29)	0.929			
Menopause status							
Post	302	ref.					
Pre	111	1.36	(1.08–1.71)	0.009 **	1.34	(1.06–1.69)	0.013 *
Abortion							
≥2	73	ref.					
0–1	340	1.33	(1.02–1.73)	0.034 *			
History of breast surgery							
No	378	ref.					
Yes	35	1.61	(1.11–2.34)	0.012 *	1.64	(1.13–2.39)	0.010 *
Use of hormonal contraceptives							
No	343	ref.			ref.		
<5 years	62	0.93	(0.70–1.24)	0.629	0.95	(0.71–1.26)	0.719
≥5 years	8	0.42	(0.20–0.86)	0.018 *	0.42	(0.21–0.87)	0.019 *
Breast composition BI–RADS							
A	0	0.00	(0.00–0.00)	0.998			
B	9	0.64	(0.30–1.35)	0.236			
C	365	1.07	(0.76–1.51)	0.703			
D	39	ref.					

Logistic regression. * *p* < 0.05, ** *p* < 0.01. Abbreviation: Num, number; OR, odds ratio; CI, confidence interval. All available risk factors were entered into the univariate analysis, but only potential ones (*p*-Value < 0.100) were listed.

## Data Availability

The dataset generated and analyzed during the current study are not publicly available but are available from the corresponding author on reasonable request.
